# Cardiac magnetic resonance feature tracking for quantifying right ventricular deformation in type 2 diabetes mellitus patients

**DOI:** 10.1038/s41598-019-46755-y

**Published:** 2019-07-31

**Authors:** Bi-yue Hu, Jin Wang, Zhi-gang Yang, Yan Ren, Li Jiang, Lin-jun Xie, Xi Liu, Yue Gao, Meng-ting Shen, Hua-yan Xu, Ke Shi, Zhen-lin Li, Chun-chao Xia, Wan-lin Peng, Ming-yan Deng, Hong Li, Ying-kun Guo

**Affiliations:** 1 Department of Radiology, West China Hospital, Sichuan University, 37# Guo Xue Xiang, Chengdu, Sichuan 610041 China; 2 Department of Radiology, Key Laboratory of Birth Defects and Related Diseases of Women and Children of Ministry of Education, West China Second University Hospital, Sichuan University, Chengdu, China; 3 Department of Endocrinology and Metabolism, West China Hospital, Sichuan University, 37# Guo Xue Xiang, Chengdu, Sichuan 610041 China; 4 Key Laboratory of Birth Defects and Related Diseases of Women and Children of Ministry of Education, West China Second University Hospital, Sichuan University, Chengdu, 610041 China

**Keywords:** Cardiology, Type 2 diabetes

## Abstract

To determine the feasibility of deformation analysis in the right ventricle (RV) using cardiovascular magnetic resonance myocardial feature tracking (CMR-FT) in type 2 diabetes mellitus (T2DM) patients. We enrolled 104 T2DM patients, including 14 with impaired right ventricular ejection fraction (RVEF) and 90 with preserved RVEF, and 26 healthy controls in this prospective study. CMR was used to determine RV feature-tracking parameters. RV strain parameters were compared among the controls, patients with preserved and reduced RVEF. Binary logistic regression was used to predict RV dysfunction. Receiver operating characteristic analysis was used to assess the diagnostic accuracy. The agreement was tested by Bland–Altman analysis. Compared with controls, longitudinal and circumferential global peak strain (PS) and PS at mid-ventricular, apical slices were significantly decreased in T2DM patients with or without reduced RVEF (p < 0.05). Within the T2DM patients, the global longitudinal PS (GLPS) and the longitudinal PS at mid-ventricular segments were significantly reduced in the reduced RVEF group than in preserved RVEF groups (p < 0.05). GLPS was an independent predictor of RV dysfunction (odds ratio: 1.246, 95% CI: 1.037–1.496; p = 0.019). The GLPS demonstrated greater diagnostic accuracy (area under curve: 0.716) to predict RV dysfunction. On Bland-Altman analysis, global circumferential PS and GLPS had the best intra- and inter-observer agreement, respectively. In T2DM patients, CMR-FT could quantify RV deformation and identify subclinical RV dysfunction in those with normal RVEF. Further, RV strain parameters are potential predictors for RV dysfunction in T2DM patients.

## Introduction

The prevalence of diabetes mellitus is increasing, with the estimated number of affected adults growing to 693 million by 2045 from 451 million in 2017 globally^1^. Diabetes mellitus patients have an increased risk of cardiovascular diseases. Cardiovascular complications remain a key cause of diabetes-related morbidity and mortality^2^. On the heart, the unfavorable influence of diabetes inevitably causes myocardial dysfunction which, initially clinically silent, might then lead to overt diabetic cardiomyopathy if early treatment is insufficient^3^. Diabetic cardiomyopathy is characterized by the occurrence of ventricular dysfunction independent of coronary artery disease and hypertension^4^. The proposed metabolic impairments contributing to diabetic cardiomyopathy include deposition of advanced glycation end products, atherosclerosis, subclinical microinfarctions, mitochondrial dysfunction, and lipotoxicity^5^. These impairments not only lead to left ventricle (LV) impairment but also might inevitably hamper right ventricle (RV) function because of the systematic nature of these impairments^6,7^.

RV function is known to have diagnostic and prognostic values in multiple cardiovascular diseases and pulmonary disorders^8,9^. The association between RV dysfunction with aggravation of myocardial function and prognosis in distinct cardiac diseases has been confirmed^10^. The favorable effect of healthy diet and physical activity on RV mechanics indicates that RV myocardial abnormalities are probably modifiable through adequate interventional strategies^11^. Most previous studies on myocardial dysfunction in diabetes have paid more attention to the LV^12,13^. The role of the RV in diabetic cardiomyopathy is still under-investigated. Cardiovascular magnetic resonance (CMR) has been regarded as the golden standard for the accurate quantification of RV function and chamber size compared with the echocardiographic assessment because of the complex anatomical and contraction patterns of the RV^8,14,15^. Further, the accuracy of echocardiographic results depends substantially on operator skill and is limited by narrow acoustic windows. There were several echocardiographic studies focused on the RV strain in T2DM patients^3,14,15^. In recent, cardiac magnetic resonance myocardial feature tracking (CMR-FT) has been treated as a novel tool with high accuracy for quantitatively assessing left and right ventricular myocardial deformation^16–18^. However, to the best of our knowledge, the information of CMR-FT for assessing the RV deformation is limited. Therefore, this study aimed to evaluate the feasibility of using CMR-FT for quantifying global and regional RV myocardial deformation and to test whether CMR-FT can detect subclinical RV dysfunction in T2DM patients.

## Methods

### Study population

This study was approved by the Institutional Ethics Review Board of West China Hospital (No. 2016-24) and we pledged to abide by the declaration of Helsinki (2000 EDITION) in accordance with the relevant medical research rules in the study. All participants provided written informed consent prior to study commencement. For this prospective study, we recruited 128 consecutive T2DM patients visiting the outpatient Department of Endocrinology and Metabolism at our institution and undergoing routine CMR examination between June 2016 and July 2018. The inclusion criteria included an established T2DM diagnosis based on the American Diabetes Association criteria^19^; no symptoms, signs, or history of heart disease (known coronary artery disease, cardiomyopathy, or valvular heart disease); sinus rhythm; and no contraindications to MR imaging. Subjects were excluded if they had severe renal impairment (estimated glomerular filtration rate <30 mL/min/1.73 mm^2^; n = 6), uncontrolled blood pressure at rest (systolic blood pressure > 180 mmHg and/or diastolic blood pressure > 100 mmHg; n = 6), or poor CMR image quality (n = 12). Known coronary heart disease included self-reported prior myocardial infarction, percutaneous coronary artery intervention, and/or coronary artery bypass grafting. Consequently, 104 T2DM patients (mean age, 52 years; range 25–77 years; male mean age, 55 years; range 29–77 years; female mean age, 54 years; range 25–74 years; P = 0.63, independent-samples t-test) remained. Patients were divided into two groups according to the recent task force criteria^20^: reduced right ventricular ejection fraction (RVEF) group (RVEF < 45%) and preserved RVEF group (RVEF ≥ 45%). Concurrently, 26 individuals from our healthy volunteer database, with similar sex and age distribution to those of our patients, were recruited to constitute the control group.

### CMR imaging

All participants underwent CMR imaging in the supine position using a 3.0-T whole-body scanner (Skyra; Siemens Medical Solutions) fitted with an 18-element body phased array coil for signal detection. We used the manufacturer’s standard ECG-triggering device and the end-inspiratory breath-holding technique to monitor ECG values and breathing, respectively. Data were acquired during the breath-holding period. In the short-axis view, 8–12 continuous CMR cine images were obtained from the level of the mitral valve to the apex using steady-state free-precession sequences (field of view [FOV] 340 × 284 mm^2^; repetition time (TR) 39.3 ms (echo spacing 2.8 ms); echo time (TE) 1.2 ms; slice thickness 8.0 mm; flip angle 40°; temporal resolution 25–40 ms; matrix size 208 × 174; voxel size 1.6 × 1.6 × 8 mm; bandwidth 1145 Hz/Px) with generalized autocalibrating partial parallel acquisition (GRAPPA, acceleration factor: 3) reconstruction. The horizontal four-chamber view cine series were also acquired.

### CMR data analysis

**Figure 1:**
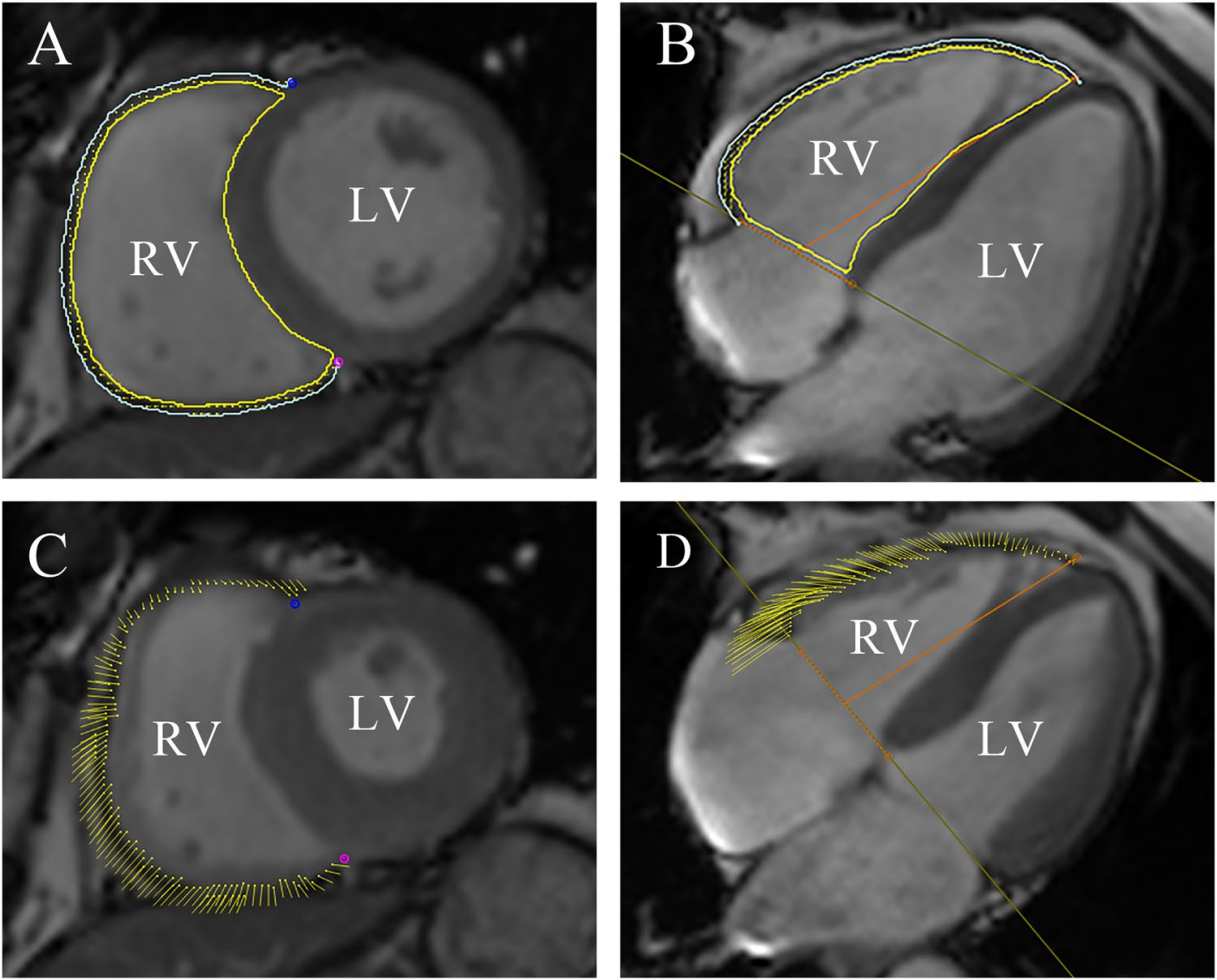
CMR feature tracking using cmr42 (Circle Cardiovascular Imaging Inc., Calgary, Canada) in short-axis, and four-chamber long-axis cine images at the end-diastole (**A**,**B**) and end-systole (**C**,**D**). The yellow and cyan curves delineate the endocardial and epicardial contours, respectively. The yellow dots represent the right ventricle myocardial voxel points, and the yellow short line on the images shows the tracking of the ventricle myocardial voxel points. The orange line is used to define the base and apex of tricuspid valve plane, and the apical plane. Abbreviations: CMR, cardiovascular magnetic resonance; T2DM: type 2 diabetes mellitus.

All CMR data were uploaded and analyzed offline using dedicated commercial software (cvi42; Circle Cardiovascular Imaging, Inc.). Cvi42 is based on an incompressible volume-based algorithm, which has been validated previously to perform accurate biventricular anatomical tracking^21^. In the serial short-axis slices, the endocardial and epicardial borders were manually outlined at the end-diastolic and end-systolic phases by an experienced radiologist (with 3 years of experience in CMR). Global parameters of RV geometry and function, including RV end-diastolic volume, RV end-systolic volume, RV stroke volume (SV), and RVEF, were computed based on serial short-axis slices; similar parameters for the LV were also computed. Both long-axis four-chamber and short-axis slices were loaded into the feature-tracking module for analyzing RV myocardial strain. In all series, the endocardial and epicardial contours were delineated manually in each slice at the end-diastole phase (reference phase), and the papillary muscles and moderator bands were carefully excluded (Fig. 1). The accuracy of feature tracking for both RV endocardial and epicardial contours was visually checked following automated strain analysis on the CMR-FT model, and good quality tracking was gained in all subjects following a maximum of two observer adjustments. Subsequently, the following three-dimenasional (3D) feature-tracking parameters were automatically acquired: longitudinal, circumferential, and radial peak strain (PS), defined as the absolute value of maximum strain measured over an entire cardiac cycle; peak diastolic strain rate (PDSR), defined as the absolute value of the maximum strain rate over all phases starting from the end systole until the end diastole; and peak systolic strain rate (PSSR), defined as the absolute value of the maximum strain rate over all phases starting from the end diastole until the end systole.

### Reproducibility

Intra-observer variability in RV strain parameters was calculated by an experienced investigator by comparing the measurements from 20 randomly selected cases analyzed by the same observer after an interval of 1 month. Inter-observer variability was estimated by comparing the measurements with those calculated by another independent double-blinded experienced observer (with more than 3 years of experience in CMR).

### Statistical analysis

Statistical analyses were performed using SPSS statistics software for MAC (version 24.0; SPSS Institute, Inc.). Normality was assessed using Kolmogorov–Smirnov test. Normally distributed data are presented as mean ± standard deviation for continuous variables and as frequencies (percentage) for categorical values. Non-parametric variables are expressed as median (interquartile range, 25–75%). One-way ANOVA or the Kruskal-Wallis test was used to assess the differences in continuous variables among the three groups. The chi-square or Fisher exact test was performed for categorical values. Spearman’s and Pearson’s correlation coefficients were used for nonparametric and normally distributed data, respectively. Binary logistic regression was used to identify predictors of RV dysfunction. Receiver operating characteristic analysis was performed to determine optimal cut-off values for RV strain parameters to identify RV dysfunction in T2DM patients. The Bland–Altman analysis was used to assess the inter- and intra-observer variabilities between acquisitions by calculating the bias (mean difference) and the 95% limits of agreement (1.96 standard deviations around the difference). Two-tailed *p* values of <0.05 were considered statistically significant.

## Results

### Baseline characteristics and biventricular geometry and function

All 142 subjects (116 T2DM patients and 26 healthy subjects) underwent CMR imaging; data sets of 104 T2DM patients and 26 healthy subjects were included in the analysis. We divided the T2DM patients into a preserved RVEF group (RVEF ≥ 45%) and a reduced RVEF group (RVEF < 45%). Data on baseline characteristics, biventricular dimensions, and cardiac functional parameters of T2DM patients and healthy subjects are presented in Table 1. Of the 104 T2DM patients, 14 (13.46%) patients presented with impaired systolic function (RVEF < 45%). Two and eight patients in the impaired RVEF group and preserved RVEF group had LV dysfunction (left ventricular ejection fraction (LVEF) < 55%), respectively. T2DM patients with or without reduced RVEF had significantly higher systolic blood pressure, diastolic blood pressure, glycosylated hemoglobin, fasting blood glucose, and high-density lipoprotein than healthy controls (*p* < 0.05). T2DM patients with reduced RVEF had significantly higher RV end-systolic volume and lower RV SV values than normal subjects or T2DM patients with preserved RVEF (*p* < 0.05).

**Table 1:** Baseline differences of clinical, biomedical, and CMR characteristics between control subjects, T2DM patients with preserved and impaired RVEF.

Variable	Control subjects (n = 26)	DM with preserved RVEF (n = 90)	DM with reduced RVEF (n = 14)
Age (years)	53 ± 10	55 ± 11	53 ± 11
Male, n (%)	16 (61.5%)	55 (61.1%)	11 (78.6%)
Height (cm)	162.0 ± 7.4	163.9 ± 8.3	166.8 ± 7.5
Weight (kg)	61.2 ± 8.8	64.6 ± 9.4	65 ± 11
Body mass index (kg/m^2^)	23.2 ± 2.3	24.0 ± 2.8	23.1 ± 3.1
Systolic blood pressure (mmHg)	118.1 ± 7.9	131 ± 15*	128 ± 12*
Diastolic blood pressure (mmHg)	78.7 ± 8.0	80 ± 11	81.6 ± 8.4
Heart rate (beats/min)	73.0 ± 9.5	76 ± 11	71 ± 12
Duration of diabetes (years)	NA	8.0 ± 6.6	6.5 ± 5.9
HbA1c (%)	5.38 ± 0.39	7.6 ± 2.2*	7.8 ± 2.1*
Fasting blood glucose (mmol/L)	5.14 ± 0.42	8.6 ± 3.9*	9.2 ± 3.5*
Plasma triglycerides (mmol/L)	1.4 ± 1.2	1.44 ± 0.81	1.41 ± 0.55
Total cholesterol (mmol/L)	4.24 ± 0.84	3.8 ± 1.7	3.9 ± 1.8
High-density lipoprotein (mmol/L)	1.28 ± 0.33	1.9 ± 1.2*	2.5 ± 2.0*
Low-density lipoprotein (mmol/L)	2.61 ± 0.61	2.30 ± 0.99	2.17 ± 0.89
Plasma creatinine (mmol/L)	63 ± 16	55 ± 33	52 ± 45
eGFR (mL/min/1.73 m^2^)	100 ± 12	93 ± 26	82 ± 21
LVEDV (ml)	128 ± 27	120 ± 27	120 ± 19
LVESV (ml)	53 ± 20	45 ± 14	47 ± 12
LVSV (ml)	75 ± 19	75 ± 17	73 ± 13
LVEF (%)	58.6 ± 9.0	62.8 ± 6.4	61.3 ± 6.9
LV myocardial mass (g)	83 ± 26	91 ± 30	89 ± 22
RVEDV (ml)	100 ± 32	102 ± 28	99 ± 17
RVESV (ml)	48 ± 16	43.7 ± 1.5	60.1 ± 8.9*^§^
RVSV (ml)	53 ± 18	58 ± 18	39 ± 11*^§^
RVEF (%)	52.5 ± 7.1	56.8 ± 6.5	38.5 ± 7.7*^§^
RV myocardial mass (g)	18.6 ± 6.1	17.4 ± 6.6	18.4 ± 3.6

Notes: The values are the mean ± SD, Numbers in the brackets are percentages.

*P < 0.05 vs. normal group; ^§^P < 0.05 vs. T2DM with normal RVEF. One-way ANOVA test was used to assess the differences in continuous variables among the three groups. The chi-square test was performed for categorical values.

DM, diabetes mellitus; RVEF, right ventricular ejection fraction; BSA, body surface area; HbA1c, glycosylated hemoglobin; eGFR, estimated glomerular filtration rate; LV, left ventricular; RV, right ventricular; EDV, end diastolic volume; ESV, end systolic volume; EF, ejection fraction; SV, stroke volume.

### Global and regional strain analysis in T2DM patients and normal controls

**Figure 2:**
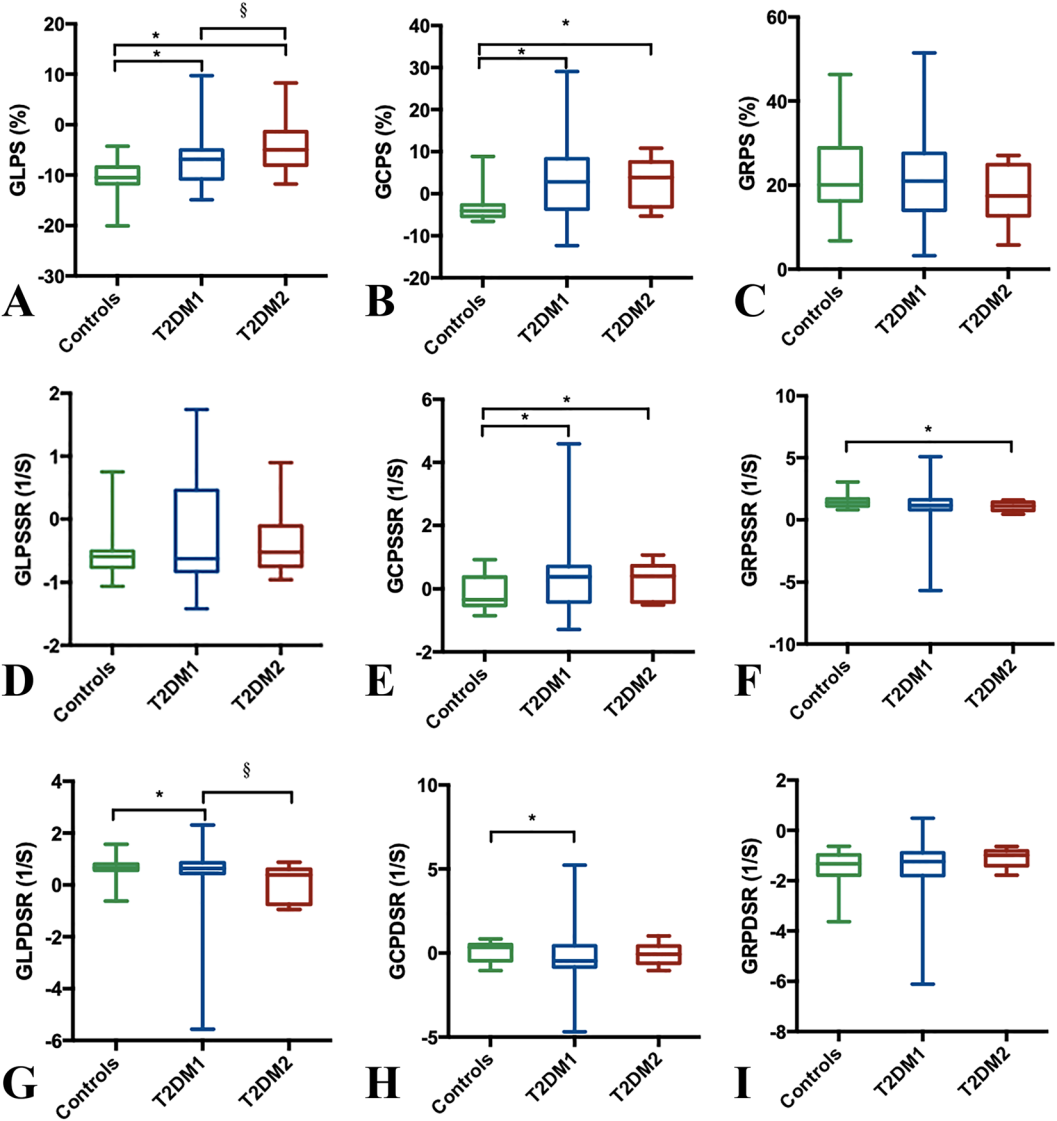
Box-and-whisker plot for comparison of global longitudinal PS, PSSR, PDSR (**A**,**D**,**G**), circumferential PS, PSSR, PDSR (**B**,**E**,**H**), and radial PS, PSSR, PDSR (**C**,**F**,**I**) in control subjects versus T2DM patients with preserved (T2DM1) and impaired RVEF (T2DM2). Kruskal-Wallis test was used to assess the differences in continuous variables among the three groups. Box plot: box length represents the interquartile range; horizontal box line represents the median; whiskers represent the maximum and minimum values; asterisk denotes P < 0.05 compared to controls; section denotes P < 0.05 T2DM compared to T2DM patients with normal RVEF. T2DM indicates, type 2 diabetes mellitus; RVEF, right ventricular ejection fraction; PS, peak strain; PSSR, peak systolic strain rate; PDSR, peak diastolic strain rate.

Data on RV global strain parameters for all participants are shown in Fig. 2. The magnitude of both global longitudinal PS (GLPS) and global circumferential PS (GCPS) were significantly decreased in T2DM patients, with or without reduced RVEF, than in healthy subjects (*p* < 0.05). Among T2DM patients, the magnitude of GLPS was significantly reduced in the reduced RVEF group than in the preserved RVEF group [−5.00(−8.00–(−1.40))% vs. −6.85(−10.78–(−5.02))%; *p* < 0.05]. There was a strong trend toward decreased magnitude of global longitudinal PDSR of T2DM patients with decreased RVEF than that of healthy subjects and T2DM patients with preserved RVEF (*p* < 0.05). The magnitude of Global radial PSSR in T2DM patients with reduced RVEF was reduced compared to that in normal subjects, while the global circumferential PSSR was higher in T2DM patients with or without preserved RVEF than that in healthy subjects (*p* < 0.05).

Data on regional RV deformation parameters among T2DM patients and healthy subjects are provided in Table 2. Individual-slice analysis showed that the magnitude of PS (longitudinal and circumferential) was significantly decreased at mid-ventricular and apical slices in T2DM patients with or without reduced RVEF than in healthy subjects (*p* < 0.05). The magnitude of longitudinal PDSR at mid-ventricular segments in T2DM patients with or without systolic RV dysfunction, as well as the longitudinal PDSR at apical slices in T2DM patients with reduced RVEF, was significantly reduced than that of controls (*p* < 0.05). Among the T2DM patients, the magnitude of longitudinal PS and PDSR at mid-ventricular segments were significantly decreased in the reduced RVEF group relative to the preserved RVEF group [0.73(−8.62–7.22)% vs. −7.69(−11.81–(−3.89))%; −0.22(−1.00–0.54) 1/s vs. 0.68(−0.27–0.90) 1/s; *p* < 0.05, respectively]. Interestingly, the magnitude of circumferential PS and PDSR at basal segments were significantly higher in T2DM patients with preserved RVEF than in healthy subjects (*p* < 0.05).

**Table 2:** Values for Right ventricular deformation parameters obtained using feature tracking for slice values of all study groups (basal, mid-ventricular, apical).

	Basal			Mid-ventricular			Apical		
	Healthy Controls (n = 26)	DM with preserved RVEF (n = 90)	DM with reduced RVEF (n = 14)	Healthy Controls (n = 26)	DM with preserved RVEF (n = 90)	DM with reduced RVEF (n = 14)	Healthy Controls (n = 26)	DM with preserved RVEF (n = 90)	DM with reducedvRVEF (n = 14)
**Longitudinal**									
PS (%)	−10.48 (−11.74–(−8.41))	−6.83 (−10.18–9.43)	−6.95 (−8.36–9.43)	−11.27 (−13.57–(−8.86))	−7.69 (−11.81–(−3.89))*	0.73 (−8.62–7.22)^§^*	−14.87 (−17.30–(−13.21))	−11.97 (−14.25–(−7.63))*	−9.88 (−13.13–(−5.38))*
PSSR (1/s)	−0.58 (−0.77–0.82)	−0.56 (−0.92–1.25)	−0.83 (−1.02–0.94)	−0.67 (−0.95–(−0.49))	−0.71 (−0.95–0.54)	−0.14 (−0.80–0.72)	−0.86 (−1.03–(−0.71))	−0.84 (−1.05–(−0.63))	−0.75 (−0.98–(−0.22))
PDSR (1/s)	0.64 (0.23–0.97)	0.63 (−1.05–1.04)	0.59 (−1.01–0.77)	0.92 (0.58–1.09)	0.68 (−0.27–0.90)*	−0.22 (−1.00–0.54)^§^*	0.92 (0.73–1.13)	0.86 (0.69–1.09)	0.66 (0.26–0.95)*
**Circumferential**									
PS (%)	10.90 (5.77–14.58)	14.26 (8.23–20.47)*	12.90 (5.09–25.35)	−7.15 (−8.71–(−4.99))	−3.83 (−6.16−7.14)*	−3.71 (−6.27–4.62)*	−10.55 (−12.93–(−6.86))	−7.08 (−11.06–(−1.00))*	−6.66 (−8.19–(−2.44))*
PSSR (1/s)	0.68 (0.44–1.12)	1.02 (0.53–1.51)	0.80 (0.52–1.95)	−0.55 (−0.69–(−0.35))	1.02 (0.53–1.51)*	−0.51 (−0.64–0.44)	−0.71 (−0.94–(−0.42))	−0.63 (−0.89–0.48)	−0.53 (−0.70–0.39)
PDSR (1/s)	−0.83 (−1.01–(−0.56))	−1.09 (−1.65–(−0.70))*	−0.97 (−1.23–0.82)	0.54 (0.36–0.84)	0.28 (−0.84–0.61)*	0.37 (−0.48–0.58)	0.63 (0.51–0.87)	0.66 (−0.49–0.92)	0.66 (0.47–0.84)
**Radial**									
PS (%)	39.98 (28.33–50.51)	38.89 (25.46–54.71)	18.52 (11.49–26.30)	22.83 (15.40–29.45)	18.70 (11.07–28.57)	18.52 (11.48–26.30)	9.23 (−2.67–16.68)	8.84 (−2.82–13.20)	7.64 (−3.17–11.08)
PSSR (1/s)	2.28 (1.68–2.62)	2.03 (1.34–2.97)	1.80 (1.18–2.37)	1.44 (1.03–1.68)	1.08 (0.77–1.64)*	1.37 (0.92–1.70)	0.92 (0.45–1.31)	0.88 (0.44–1.24)	0.72 (0.52–0.91)
PDSR (1/s)	−2.30 (−3.40–(−1.76))	−2.17 (−3.22–(−1.60))	−2.00 (−2.79–(−1.62))	−1.61 (−1.89–(−1.08))	−1.21 (−1.59–(−0.86))*	−1.11 (−1.59–0.85)	−0.87 (−1.35–(−0.43))	−0.92 (−1.48–(−0.40))	−0.85 (−1.08–(−0.34))

Notes: Data are presented as the median (25th, 75th percentile).

*P < 0.05 vs. normal group. ^§^P < 0.05 vs. T2DM with normal RVEF. The Kruskal-Wallis test was used to assess the differences in continuous variables among the three groups. DM, diabetes mellitus; RVEF, right ventricular ejection fraction; PS, peak strain; PSSR, peak systolic strain rate; PDSR, peak diastolic strain rate.

### Correlations of RV global strain parameters with RV and LV dimensional and functional parameters and biochemical markers

**Figure 3:**
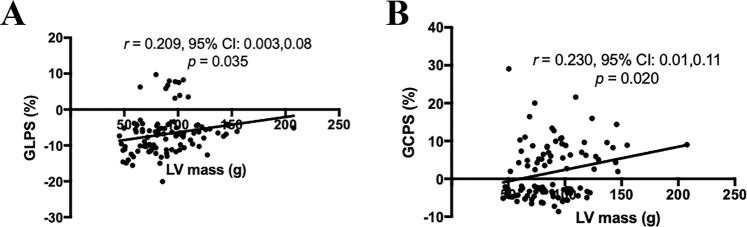
Scattergrams show the results of Pearson correlation analysis between LV mass and global (**A**) longitudinal, and (**B**) circumferential peak strain measurements.

The associations of the global strain parameters with HbA1c, fasting blood glucose, plasma triglycerides, total cholesterol, high-density lipoprotein, low-density, lipoprotein, plasma creatinine, estimated glomerular filtration rate, LV end diastolic volume (EDV), LV end systolic volume (ESV), LVSV, LVEF, LV and RV myocardial mass, RVEDV, RVESV, RVSV, and RVEF are tested. The significant associations are shown in Fig. 3. RV GLPS was correlated to LV mass (r = 0.209, *p* = 0.035). RV GCPS was associated with LV mass (r = 0.230, *p* = 0.020).

### Predictors of RV dysfunction

As shown in Table 3, a binary logistic regression model that used RV dysfunction (RVEF < 45%) as the dependent variable identified GLPS as an independent predictor of RV dysfunction (odds ratio: 1.246, 95% CI: 1.037–1.496; *p* = 0.019), adjusted for age, sex, body mass index, glycosylated hemoglobin, high-density lipoprotein, global circumferential PS, and global radial PS.

**Table 3:** Univariate and multivariable stepwise backward logistic regression analysis for the prediction of RV dysfunction (n = 130).

Variable	Univariate		Multivariate		
	R	P value	Odds ratio	95% CI	P value
Age (years)	−0.055	0.533	0.975	0.906–1.049	0.497
Sex (male)	0.112	0.206	0.051	0.002–1.238	0.067
BMI (kg/m^2^)	−0.082	0.357	0.878	0.639–1.206	0.421
Height (cm)	0.127	0.150			
Weight (kg)	0.022	0.807			
Systolic blood pressure (mmHg)	0.012	0.904			
Diastolic blood pressure (mmHg)	0.056	0.563			
Heart rate (beats/min)	−0.108	0.225			
Duration of diabetes (years)	−0.077	0.479			
HbA1c (%)	0.112	0.242	1.160	0.857–1.570	0.337
Fasting blood glucose (mmol/L)	0.151	0.159			
Plasma triglycerides (mmol/L)	−0.006	0.957			
Total cholesterol (mmol/L)	−0.002	0.986			
High-density lipoprotein (mmol/L)	0.199	0.042*	1.332	0.805–2.205	0.264
Low-density lipoprotein (mmol/L)	–0.073	0.460			
Plasma creatinine (mmol/L)	−0.052	0.595			
eGFR (mL/min/1.73 m^2^)	−0.085	0.419			
GLPS (%)	0.222	0.011*	1.246	1.037–1.496	0.019*
GCPS (%)	0.049	0.583	0.973	0.859–1.104	0.674
GRPS (%)	−0.135	0.126	0.978	0.899–1.064	0.606

Notes: GCPS, global circumferential peak strain; GRPS, global radial peak strain; GLPS, global longitudinal peak strain; HbA1c, glycosylated hemoglobin; eGFR, estimated glomerular filtration rate; BMI, body mass index.

### Analysis of diagnostic performance

**Figure 4:**
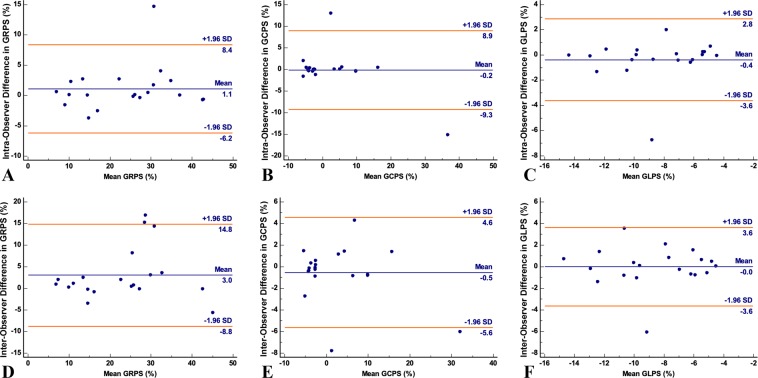
Bland–Altman plots with limits of agreement (95% confidence intervals) demonstrating the intra-observer (**A**–**C**) and inter-observer (**D**–**F**) reproducibility of CMR myocardial feature tracking strain parameters: GRPS = global radial peak strain; GCPS = global circumferential peak strain; GLPS = global longitudinal peak strain. Solid lines represent bias (blue) and 95% limits of agreement (orange).

Receiver operating characteristic (ROC) analysis showed the predictive value of RV strain parameters, including global longitudinal, circumferential, and radial PS, for RV dysfunction in T2DM patients (Fig. 4). The area under the ROC curve and sensitivity and specificity of the RV global strain parameters used for the discrimination of RV deformation between patients and controls are summarized in Table 4.

**Table 4:** Inter- and intra-observer variability of tissue tracking (n = 20).

	Inter-observer variability		Intra-observer variability	
	Mean bias ± SD	95%CI	Mean bias ± SD	95%CI
GLPS (%)	−0.39 ± 1.65	−3.62–2.85	−0.02 ± 1.85	−3.64–3.61
GCPS (%)	−0.19 ± 4.64	−9.28–8.90	−0.54 ± 2.6	−5.63–4.56
GRPS (%)	1.08 ± 3.71	−6.19–8.35	3.00 ± 6.03	−8.81–14.81

Note: 95%CI, 95% Confidence interval. GRPS = global radial peak strain; GCPS = global circumferential peak strain; GLPS = global longitudinal peak strain.

### Inter-observer and intra-observer variabilities

**Figure 5:**
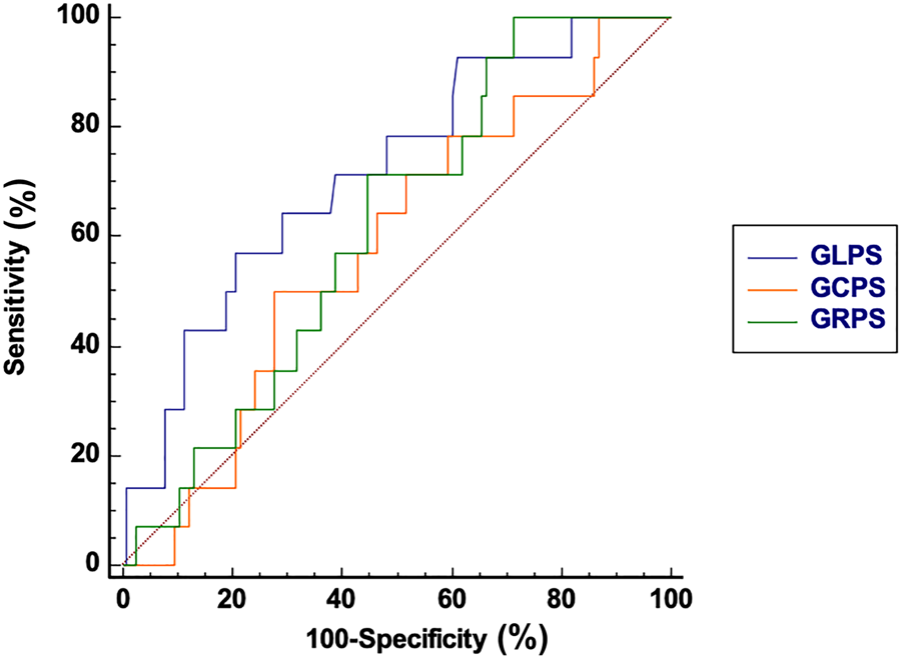
ROC analysis of global longitudinal PS (blue), circumferential PS (orange), and radial PS (green) between patients with T2DM and controls. Abbreviations: T2DM, type 2 diabetes mellitus; PS, peak strain; ROC, receiver operating characteristic.

The Bland–Altman analysis indicated moderate-to-excellent agreement between intra- and inter-observer acquisitions in measuring global radial, circumferential, and longitudinal PS (Fig. 5). The mean bias ± standard deviations and 95% confidence interval of the Bland–Altman analysis are summarized in Table 5.

**Table 5:** ROC analysis of CMR feature tracking for detecting RV dysfunction between T2DM patients and normal controls.

	Cut off	AUC	Sensitivity (%) (95%CI)	Specificity (%) (95%CI)
Global Longitudinal PS (%)	−5.14	0.716	57.14 (28.9–82.2)	79.31 (70.8–86.3)
Global Circumferential PS (%)	5.23	0.579	50.00 (23.1–76.9)	72.41 (63.3–80.3)
Global Radial PS (%)	27.05	0.617	100.00 (76.7–100.0)	28.45 (20.5–37.6)

Notes: ROC, receiver operating characteristic; AUC, area under the ROC curve; CI, confidence interval. PS, peak strain.

## Discussion

In this study, we describe the results of a prospective study on detailed RV global and regional myocardial strain analysis in adult T2DM patients using CMR-FT. To the best of our knowledge, this is the first report of its kind. We demonstrated that CMR-FT can be used to quantify RV myocardial deformation in T2DM patients and that these measurements can provide objective evaluations of subclinical RV dysfunction before clinically manifested reduction in RVEF; specifically, we showed that GLPS has the greatest diagnostic potential among the tested RV feature-tracking parameters. Further, we concluded that GLPS might provide additional information in predicting RV dysfunction in T2DM patients.

Recently, CMR-FT has emerged as a novel tool to quantitatively assess biventricular myocardial function^16–18^. CMR-FT utilizes tissue voxel motion-tracking technology based on CMR cine images. As its main advantage, it has a relatively simple post-processing procedure. Further, the estimated 10-min post-processing time for each subject implies that this method can be used as a routine procedure^22–24^. Intra-observer reproducibility of CMR-derived strain analysis does not depend on the magnetic field strength^25^, and in our study, both intra- and inter-observer reproducibilities for global PS were excellent. Further, the reproducibilities of the radial PS measurements were better than that reported previously^26^. Previous CMR-FT studies using two-dimensional imaging model have shown that normal RV strain was almost twice as much as ours; the reason is that our study was based on 3D CMR-FT model with totally different algorithm^27,28^.

RV function is often difficult to evaluate because of its complex shape and motion. CMR imaging is indeed regarded as the gold standard for assessing RV dimension and function. However, because of the complex RV contraction patterns, regional wall motion abnormalities are still inadequately analyzed. Measures of myocardial deformation, such as strain analysis, are emerging modalities to quantitatively assess regional and global RV function^29^. The usefulness of myocardial strain imaging to quantify RV function in T2DM has been previously demonstrated using tissue Doppler and speckle-tracking echocardiography studies, which consistently showed reduced RV myocardial strain in T2DM patients compared with that in healthy subjects. However, these studies were of limited value in the exact quantification of RV function and chamber size^14,30–32^. However, despite the large volume of information on echocardiographic imaging data, information on the applicability of deformation imaging in T2DM using CMR is still limited. Thus, our results provide complementary and more accurate information on detailed global and regional RV strain measurements with respect to echocardiographic data. Myocardial tagging is considered the standard or reference CMR technique to quantitatively evaluate LV regional strain; however, applying tagging lines to the thin myocardial wall of the RV and the time-consuming and complex procedure of data acquisition and post-processing have limited its popularity^4^. Conversely, based on the results of a previous study that are comparable to those obtained using CMR tagging, CMR-FT imaging is a promising technique to overcome these limitations and challenges^33^. Furthermore, recent advances in feature-tracking technology offer powerful modalities for evaluating RV contractile function and diastolic relaxation in patients without RV hypertrophy^34^. However, Wehner *et al*. reported that CMR-FT inaccurately quantifies LV strain values when compared to displacement encoding with stimulated echoes (DENSE) imaging as a reference standard. However, they only evaluated the agreement between measurements of two techniques, and they could not evaluate the prognostic utility of the measures^35^. These results could not deny the diagnostic and prognostic values of strain measurements derived from CMR-FT which has been established^9,36^. Furthermore, when applied to RV, DENSE imaging was limited by its lengthy scan time, lower resolution, difficulty to distinguish the minimum from the intermediate principal strains, and the reduction in signal to noise ratio associated with the stimulated echo^37^.

We showed impaired RV global and regional strain in T2DM patients compared with those in healthy individuals and notably the ability of GLPS to detect and predict RV dysfunction. These RV strain changes in T2DM patients seemed to precede overt RV dysfunction, as they were recognized even in patients without impaired RVEF. As described elsewhere, the deep muscle layer of the RV myocardial wall is primarily composed of longitudinal fibers^33^; this architectural pattern of the RV contributes to the predominantly longitudinal shortening that leads to blood ejection during systolic phase. Importantly, this structure can also explain the predominance of longitudinal RV strain changes observed in our study. Specifically, GLPS with optimal cut-off value of −5.14% had a higher diagnostic accuracy in identifying RV dysfunction in T2DM patients without clinical symptoms of heart failure, thus fulfilling the requirement for more sensitive CMR parameters that can be used for early diagnosis of this condition^38^. Data obtained from the ROC analysis demonstrated the diagnostic performance of global radial, circumferential, and longitudinal PS, as the criteria for differentiating RV dysfunction was moderate in this patient group. Peter *et al*. have established that the LV global longitudinal strain could be used for prediction of silent myocardial infarction which is associated with significant mortality and morbidity in T2DM patients^39^. Whether RV GLPS in diabetes is predictive of silent myocardial infarction should be clarified in the future. Interestingly, the circumferential strain at basal slices increased in T2DM patients with preserved RVEF. We hypothesized that in the early stages of diabetes, epicardial fibers remaining are spared, which compensate for the longitudinal dysfunction and thus preserve SV and ejection fraction^25^. Blood pressure control in hypertensive patients has been shown to decrease circumferential strain, which also might be helpful to explain it as our cohort included T2DM patients with higher blood pressure than controls^25^. As subclinical RV dysfunction reflects the structural and metabolic milieu of the myocardium, RV strain data might be useful to develop targeted therapeutic strategies that modulate cardiac metabolism and prevent heart failure in T2DM^40^. Besides, further studies are required to determine whether global and regional RV strain parameters, especially of GLPS, can be used as risk factors to predict outcomes in T2DM patients.

In this study, we have not found any significant correlations between the HbA1c level and RV volumes and function parameters, which does not agree with the findings of previous studies^41^. To the best of our knowledge, significant relationships between RV GLPS, GCPS, and LV mass were observed in our study, which has not been demonstrated. The concentric LV hypertrophy, defined as increased LV mass, has shown to be a strong predictor of adverse cardiovascular events in T2DM patients^7^. In our cohort, we did not observe signifancant increased LV mass in diabetes patients. We may hypothezised that impaired RV GLPS and GCPS in T2DM patients might be predictors for adverse clinical outcomes. Further follow-up studies in this patient population are warranted.

Our study has several limitations. First, this was a single-center study with a limited sample size and included only 14 subjects with impaired RVEF. Therefore, these findings need further validation using larger cohort or multi-center results. Second, several software can be used to analyze RV deformation, data on RV myocardial strain quantification using CMR-FT is insufficient. Thus, reference values for RV strain should be determined. Finally, as clinical follow-up data were not available for the study patients, the clinical correlation and prognostic implications of RV strain parameters in T2DM patients should be verified in our futher studies.

In summary, we showed that RV myocardial strain assessment can be used to identify RV deformation in T2DM patients, even in those with preserved RVEF, implying that RV strain parameters is more sensitive than RVEF values for the early detection of subclinical RV dysfunction in T2DM patients. Further, RV strain parameters may have diagnostic value for RV dysfunction and might be useful predictors of RV dysfunction in T2DM patients.

## Data Availability

The datasets used during the current study are available from the corresponding author on reasonable request.
